# Periprocedural Wound Complication Risks in Cancer Pain Patients Following Targeted Cancer Therapy: A Retrospective Analysis

**DOI:** 10.1002/cnr2.70664

**Published:** 2026-10-14

**Authors:** Soun Sheen, Vishal Bansal, Xiaowen Sun, Jian Wang, Saba Javed

**Affiliations:** ^1^ Department of Pain Medicine The University of Texas MD Anderson Cancer Center Houston Texas USA; ^2^ Department of Orthopedics and Physical Rehabilitation University of Massachusetts Memorial Medical Center Worcester Massachusetts USA; ^3^ Department of Biostatistics The University of Texas MD Anderson Cancer Center Houston Texas USA

**Keywords:** cancer pain, chronic pain, neuromodulation, wound healing

## Abstract

**Background:**

In light of the increasing use of advanced operative procedures for cancer pain, it is crucial for pain physicians to understand the unique potential risks associated with multimodal cancer treatments.

**Aims:**

We aimed to characterize the periprocedural wound healing risk associated with newer targeted cancer therapies in cancer pain patients.

**Methods and Results:**

A retrospective cohort study was conducted in patients 18 years or older undergoing operative procedures for cancer‐related pain. The treatment group comprised patients on targeted cancer therapies, and the control group comprised patients not on these treatments. A generalized estimating equations model was used to compare the risk of wound‐related complications between groups. A total of 195 encounters were identified in 150 patients. Wound‐related complications occurred in 4/44 (9.1%) encounters in the treatment group and 3/151 (2.0%) in the control group. The treatment group had higher odds of wound‐related complications (OR: 7.41, 95% CI: 1.36–40.40; *p* = 0.02). Higher BMI, diabetes, and concurrent steroid use also contributed to a higher risk of wound‐related complications.

**Conclusions:**

Our study suggests that concurrent targeted cancer therapies may be associated with an increased risk of wound‐related complications, although the estimate is imprecise given a heterogeneous cohort and a small number of events. It is essential for pain physicians to understand the potential risks associated with these cancer treatments and to collaborate with oncologists in the periprocedural period to optimize patient care.

## 1 Introduction

In light of advancing treatments for cancer and improving survival rates, there is increased emphasis on managing the secondary consequences of cancer and its treatment, including cancer‐related pain. Although pain may not be life threatening for cancer patients, it remains one of the most disabling symptoms, associated with decreased quality of life and function, increased health care utilization, and reduced adherence to cancer treatment, all of which can collectively lead to lower survival rates [1, 2, 3]. The treatment of cancer‐related pain has historically followed the stepwise analgesic ladder approach outlined by the World Health Organization (WHO) [4]. Despite high doses of opioids, about 25%–40% of cancer patients experience refractory cancer pain [5, 6, 7]. Furthermore, approximately 10%–20% of patients who are getting adequate relief from medications ultimately reduce their doses due to adverse effects, leaving them with inadequate pain relief [5]. Patients may also develop chronic cancer pain with significant neuropathic pain components often refractory to conventional medication therapy [8]. Furthermore, especially with improving cancer survival, conventional cancer pain management based on the WHO guidelines has raised concerns due to its heavy reliance on opioids, leading to increased utilization of non‐pharmacological modalities [7, 8].

In patients with refractory cancer pain, there may be a key role for interventional treatment modalities, ranging from local anesthetic and steroid injections and sympathetic neurolysis to more advanced operative neuromodulatory therapies, including intrathecal drug delivery systems (IDDS) and spinal cord stimulation (SCS) [9]. These procedures allow for more targeted treatment, tailored to each patient's pain presentation. While there is a growing body of evidence to support utilization of these operative modalities for cancer pain, there remains a lack of standardized guidelines to optimize safety specifically in cancer patients [5, 8, 10].

Given the unique consequences of cancer and cancer treatments, several factors must be considered preoperatively in cancer patients to reduce potential postprocedural complications. Specifically, wound healing is a critical factor in cancer patients, as it is a complex process often disrupted by cancer or cancer treatments. While wound‐related complications associated with a number of cancer treatments are well documented, the focus of this study is to better understand the impact of modern targeted cancer therapies.

Different types of targeted cancer therapies modulate or enhance immune activation to potentiate cancer cell destruction [11]. Although the exact mechanism of each medication class and their impact on wound healing are beyond the scope of this study, it is believed that different types of targeted cancer therapies enhance immune activation to create an immunological milieu that potentiates cancer cell destruction [11]. However, by enhancing immune responses, targeted cancer therapies may lead to excessive inflammation at the wound site, potentially delaying wound healing and increasing infection risks [11, 12]. These drugs also include agents that target various growth factors, such as vascular endothelial growth factors (VEGF), which play a crucial role in wound healing [13].

Periprocedural management recommendations vary considerably by agent. While some agents have suggested hold periods based on pharmacokinetics and proposed wound‐healing effects, others do not have specific periprocedural hold recommendations [14]. However, despite their increased use, there remains a lack of standardized guidelines for the periprocedural management of patients receiving these newer therapies. Especially as the use of operative treatment modalities has increased for cancer pain patients, it is crucial for pain physicians to understand and manage the potential risks associated with these treatments to optimize patient care. In this study, we aimed to characterize the periprocedural wound‐related risks associated with newer targeted cancer therapies in cancer pain patients. We also aimed to identify intrinsic and extrinsic factors that may affect the risk of poor wound healing.

## 2 Methods

### 2.1 Design

We conducted a retrospective cohort study of patients 18 years or older being treated at an academic, tertiary‐care comprehensive cancer center who underwent operative pain procedures, including an SCS/dorsal root ganglion stimulation (DRGS) trial procedure, SCS/DRGS implantation, SCS/DRGS revision/removal, peripheral nerve stimulation (PNS) implantation, PNS explantation, IDDS implantation, and IDDS revision/removal. All operative pain procedures were performed by a board‐certified pain physician with or without the assistance of a trainee. The treatment group comprised patients on concurrent targeted cancer therapy drugs, while the control group comprised patients not receiving these treatments. The treatment group only included patients actively receiving a targeted agent at the time of the procedure. This study was approved by the institutional review board (#2024‐0319), and the requirement for informed consent was waived, as the study was retrospective, minimal risk, and could not be practically performed without a waiver. The Strengthening the Reporting of Observational Studies in Epidemiology (STROBE) guidelines were followed for this study. While patient and public involvement is an important aspect of research, the scope of this particular study did not include direct involvement of patients or the public.

Study data was searched for within the period from March 4, 2016, to January 8, 2024, using the Epic electronic medical record system. The following demographics and clinical data were collected: age, gender, body mass index (BMI), primary cancer diagnosis, diabetes status (yes vs. no), smoking status (current vs. former vs. never), concurrent steroid use (yes vs. no), history of prior surgical site infection (yes vs. no), targeted cancer therapy drug and class, duration of holding targeted cancer therapies before and after surgery, and postprocedural wound‐related complications (yes vs. no). Wound‐related complications within 90 days of the procedure included surgical site infection, wound dehiscence, and peri‐incisional fluid collections including seroma and hematoma. Although seroma and hematoma do not uniformly represent wound healing failure, they were classified as wound‐related complications for this study. Each complication was defined as follows: surgical site infection, purulent drainage, positive wound culture, or clinical diagnosis requiring antibiotic treatment or further surgical treatment including washout; wound dehiscence, partial or complete separation of the incision; seroma, peri‐incisional fluid collection; and hematoma, a peri‐incisional blood collection. The retrieved data were manually reviewed by clinician investigators (S.S. and V.B.). Missing data were < 5% across all variables. Cases with missing data were excluded and complete case analysis was performed.

### 2.2 Analyses

Descriptive statistics were used to summarize the clinical and demographic characteristics of all patients included. Targeted cancer therapies were analyzed as a single exposure category given the small number of patients and formal subgroup analyses by drug class were not performed due to sample size. For patients who underwent multiple operative pain procedures (encounters) and hence, had multiple values collected at different time points (e.g., age, BMI), average values across all time points were used. The procedure‐related variables (e.g., steroid use, procedure, complication) were summarized at the level of the procedure. Means and standard deviations (SD) were used for continuous variables (e.g., age, BMI), and frequencies and proportions were used for categorical variables (e.g., gender, cancer type). A generalized estimating equations model with exchangeable correlation structure was used to compare the risk factors between the treatment and control groups, accounting for repeated procedures within each patient. A two‐sided *p*‐value of < 0.05 was considered statistically significant. The statistical analysis was conducted using SAS 9.4 (SAS, Cary, NC).

## 3 Results

A total of 195 encounters were identified in 150 patients who underwent operative pain procedures from March 4, 2016, to January 8, 2024. Among them, 39 patients were on concurrent targeted cancer therapy at the time of the procedures. Of the 111 patients in the control group (those not on targeted cancer therapy), 29 patients (26.1%) were on other antineoplastic treatments and 82 patients (73.9%) were not on any antineoplastic treatment at the time of the procedures. Patient characteristics are summarized in Table 1. The overall mean age was 57.8 years, and 91 (46.7%) patients were women. The mean age for the treatment group was 61.4 years, and 43.2% were women, whereas the mean age for the control group was 56.8 years, and 47.7% were women.

**TABLE 1:** Demographic and clinical characteristics (*N*, number of encounters).

Characteristic	Control (*N* = 151)	Treatment (*N* = 44)	Total (*N* = 195)	*p* ^a^
Age, mean (SD)	56.8 (16.2)	61.4 (13.3)	57.8 (15.7)	0.32
Gender, *N* (%)				0.77
Female	72 (47.7%)	19 (43.2%)	91 (46.7%)	
Male	79 (52.3%)	25 (56.8%)	104 (53.3%)	
BMI, mean (SD)	30.8 (7.2)	33.6 (8.2)	31.4 (7.5)	**0.05**
Diabetes, *N* (%)				0.88
No	117 (77.5%)	34 (77.3%)	151 (77.4%)	
Yes	34 (22.5%)	10 (22.7%)	44 (22.6%)	
Smoking, *N* (%)				
Former (ref)	76 (50.3%)	21 (47.7%)	97 (49.7%)	
Never	54 (35.8%)	18 (40.9%)	72 (36.9%)	0.78
Current	21 (13.9%)	5 (11.4%)	26 (13.3%)	0.72
Steroid use, *N* (%)				**0.01**
No	147 (97.4%)	34 (77.3%)	181 (92.8%)	
Yes	4 (2.6%)	10 (22.7%)	14 (7.2%)	
Prior post‐surgical infection, *N* (%)				0.15
No	149 (98.7%)	40 (90.9%)	189 (96.9%)	
Yes	2 (1.3%)	4 (9.1%)	6 (3.1%)	

*Note:* Bold indicates statistical significance.

Abbreviations: Ref, reference; SD, standard deviation.

^a^ Generalized estimating equations model *p*‐value.

The treatment group had a significantly higher average BMI (33.6) compared to the control group (30.8). The treatment group also had a significantly higher proportion of patients receiving concurrent oral steroid therapy at the time of the procedure (22.7% vs. 2.6% in the control group; Table 1).

There were 44 encounters identified in the treatment group: 4 DRGS trials, 3 DRGS implantations, 9 IDDS implantations, 6 IDDS revision or removal, 13 SCS trials, 5 SCS implantations, and 4 SCS removal or revision (Table 2). Among the 151 encounters in the control group, the majority involved SCS trials, implantations, and removal or revision. The documented targeted cancer therapy drugs and their classes are listed in Table 3. The most common therapies in the treatment group were Rituximab (27.3%) and pembrolizumab (18.2%).

**TABLE 2:** Procedures performed (*N*, total number of encounters).

Procedure	Control, *N* (%) (*N* = 151)	Treatment, *N* (%) (*N* = 44)	Total, *N* (%) (*N* = 195)
DRGS trial	8 (5.3)	4 (9.1)	12 (6.2)
DRGS implantation	6 (4)	3 (6.8)	9 (4.6)
DRGS revision/removal	3 (2.0)	0 (0.0)	3 (1.5)
IDDS implantation	13 (8.6)	9 (20.5)	22 (11.3)
IDDS revision/removal	20 (13.2)	6 (13.6)	26 (13.3)
PNS trial	1 (0.7)	0 (0.0)	1 (0.5)
PNS implant	10 (6.6)	0 (0.0)	10 (5.1)
PNS explant	1 (0.7)	0 (0.0)	1 (0.5)
SCS trial	34 (22.5)	13 (29.5)	47 (24.1)
SCS implantation	27 (17.9)	5 (11.4)	32 (16.4)
SCS removal/revision	28 (18.5)	4 (9.1)	32 (16.4)

Abbreviations: DRGS, dorsal root ganglion stimulation; IDDS, intrathecal drug delivery system; PNS, peripheral nerve stimulation; SCS, spinal cord stimulation.

**TABLE 3:** Targeted cancer therapy drugs in the treatment group.

Therapy drugs	Drug class	Frequency, *N* (%)
Adalimumab	TNF‐alpha inhibitor	5 (11.4)
Atezolizumab	ICI (PD‐L1)	1 (2.3)
Bevacizumab	Anti‐VEGF monoclonal antibody	2 (4.6)
Cabozantinib	VEGF TKI	2 (4.6)
Lenvatinib	VEGF TKI	3 (6.8)
Nivolumab	ICI (PD‐1)	5 (11.4)
Pazopanib	VEGF TKI	2 (4.6)
Pembrolizumab	ICI (PD‐1)	8 (18.2)
Regorafenib	VEGF TKI	1 (2.3)
Rituximab	CD20 monoclonal antibody	12 (27.3)
Combination therapy		3 (6.8)
Ipilimumab + Nivolumab	ICI (CTLA‐4) + ICI (PD‐1)	2 (4.6)
Cabozantinib + Nivolumab	VEGF TKI + ICI (PD‐1)	1 (2.3)

Abbreviations: CTLA‐4, cytotoxic T‐lymphocyte antigen‐4; ICI, immune check point inhibitor; PD‐1, programmed cell death protein 1; PD‐L1, programmed cell death ligand 1; TKI, tyrosine kinase inhibitor; VEGF, vascular endothelial growth factor.

Among the 195 encounters, there were a total of 7 wound‐related complications, with 4 cases of complications in the treatment group (9.1%) and 3 in the control group (2.0%). The treatment group had higher odds of wound‐related complications than the control group (odds ratio (OR):7.41, 95% confidence interval (CI): 1.36–40.40; *p* = 0.02) (Table 4). However, the CI range was wide due to the small number of events. In the treatment group, complications included one wound dehiscence following SCS implantation, two surgical site infections (one after SCS trial and one after DRGS trial), and one seroma following IDDS implantation. In the control group, complications included two wound dehiscences (one after an IDDS implantation and one after an IDDS explantation) and one surgical site infection following an IDDS implantation (Table 5).

**TABLE 4:** Risk of wound‐healing complications.

Covariate^a^	Odds ratio	95% CI	*p*
Treatment (ref: control)	7.41	1.36, 40.40	**0.02**
Age	1.00	0.94, 1.10	0.94
BMI	1.14	1.07, 1.22	**0.0001**
Gender (ref: female)	0.89	0.18, 4.42	0.88
Smoking (ref: former)			
Current	1.31	0.14, 12.50	0.81
Never	0.94	0.16, 5.62	0.95
Diabetes	7.66	1.41, 41.60	**0.02**
Steroid use	7.50	1.44, 39.12	**0.02**
Prior post‐surgical infection	7.39	0.89, 61.08	0.06

*Note:* Bold indicates statistical significance.

^a^ The reference used is noted if applicable.

**TABLE 5:** Wound‐related complications by group.

Complication type	Treatment group (*N* = 44)	Control group (*N* = 151)
Wound dehiscence	1 (SCS implantation)	2 (1 IDDS implantation, 1 IDDS explantation)
Surgical site infection	2 (1 SCS trial, 1 DRGS trial)	1 (IDDS implantation)
Seroma	1 (IDDS implantation)	0
Hematoma^a^	0	0
Total, *n* (%)	4 (9.1%)	3 (2.0%)

Abbreviations: DRGS, dorsal root ganglion stimulation; IDDS, intrathecal drug delivery system; SCS, spinal cord stimulation.

^a^ Hematoma shown for completeness; no events observed.

Other risk factors, including BMI, diabetes, and concurrent steroid use, were also associated with increased risk of wound‐related complications. Diabetes was associated with higher odds of wound‐related complication (OR: 7.66, 95% CI: 1.41–41.60, *p* = 0.02), again with a wide CI reflecting the small number of events. Concurrent steroid use was similarly associated with higher odds of complication (OR: 7.50, 95% CI: 1.44–39.12; *p* = 0.02). Additionally, one unit increase in BMI resulted in a 14.1% increase in the odds of developing wound‐related complications (OR: 1.14, 95% CI: 1.07–1.22; *p* = 0.0001). There was no statistically significant association with age, gender, smoking, or prior history of post‐surgical infection.

## 4 Discussion

Our study offers valuable insights into the periprocedural risks associated with the use of targeted cancer therapies in cancer pain patients. The findings suggest that patients on these treatments are at a significantly higher risk of wound‐related complications.

While targeted cancer therapies have drastically improved patient survival, this progress has also led to a growing prevalence of cancer pain and a shift toward multimodal pain management that minimizes opioid use [1, 7, 8]. Additionally, unlike conventional chemotherapy, many targeted cancer therapies also have an indefinite treatment course, meaning physicians will increasingly encounter periprocedural patients on these treatments [14, 15]. Although understanding the pharmacological profiles of these treatments may be beyond the typical scope of pain physicians, they must recognize the potential risks associated with the unique consequences of cancer and its treatments.

The absolute rate of wound‐related complications was 9.1% (4/44) in the treatment group versus 2.0% (3/151) in the control group. Although the point estimate of the OR was elevated, the wide CI and small number of events limit the precision of this estimate, precluding firm causal inference. These observations are broadly consistent with prior mechanistic and clinical reports describing the impact of targeted cancer therapies on wound healing [11, 12, 13, 14].

Despite the growing use of these agents, there is a near absence of procedure‐specific periprocedural guidance. Perioperative guidance has largely come from specialty consensus statements or narrative reviews rather than randomized data [14, 15]. Furthermore, hold recommendations differ substantially by drug class and many agents do not have specific periprocedural hold recommendations. The recent consensus guidelines provide guidance on infection prevention and anticoagulation management, but not specific to the cancer pain population.

Despite the known periprocedural risks, 40 of the 44 encounters in the treatment group (90.9%) lacked clear documentation regarding periprocedural treatment regimen modifications. Holding cancer treatment carries its own risks, including the potential for disease progression. Given all the implications of this decision, it is crucial for pain physicians to collaborate closely with oncologists to optimize patient care in the periprocedural period. Of note, communication between oncologists and pain specialists is often inadequate, particularly in outpatient settings where they may not be affiliated with the same institution.

In addition to targeted cancer therapy, our study identified several other factors that contributed to the risk of wound‐related complications, including increased BMI, diabetes, and concurrent steroid use. Obesity, diabetes, and corticosteroid use are each established impediments to wound healing through multiple well‐characterized mechanisms, including impaired tissue perfusion and microvascular dysfunction, chronic inflammation, altered growth‐factor signaling, and greater mechanical stress at surgical sites [16, 17, 18]. These factors were unevenly distributed in our cohort—the treatment group had significantly higher mean BMI (33.6 vs. 30.8) and higher rates of concurrent steroid use (22.7% vs. 2.6%). Given these imbalances, coupled with the small number of events, a meaningful portion of the apparent excess risk attributed to targeted cancer therapy may be confounded and overestimated. There was no significant association between wound‐related complications and the other variables examined. Further investigation into the impact of various intrinsic and extrinsic factors specific to cancer patients in a larger, prospective study is warranted.

Cancer pain management is challenging given the heterogeneous nature of pain presentations, varying comorbidities, and complication risks in cancer patients, which may limit the use of certain pain treatment modalities. Our findings highlight the need for the development of evidence‐based guidelines to ensure patient safety in this population. These guidelines should address not only the management of periprocedural cancer treatments, but also other patient‐specific factors that may be unique to cancer patients. Pain physicians should also explore alternative non‐operative treatment modalities when necessary to minimize the risk of complications.

This study has several limitations. Due to the nature of retrospective studies, there are unquantifiable sources of bias that may have influenced the results. Only 39 of 150 patients (44 of 195 encounters) received targeted cancer therapy, and a total of only seven complications were observed overall. This resulted in wide CI and a substantial risk of overfitting in the multivariable model. The reported odds ratio should therefore be interpreted with caution, and causal inferences cannot be drawn. Furthermore, there is a large documentation gap. Notably, the majority of the treatment group encounters lacked explicit documentation of whether the targeted cancer therapy agents were held, continued, or adjusted periprocedurally.

Due to the small sample sizes, we included all advanced interventions for cancer pain, resulting in a procedurally heterogenous cohort. We recognize that these interventions differ substantially in depth and are not clinically equivalent. As the primary aim of this study was to characterize overall periprocedural wound‐related complications in cancer pain patients, procedure‐specific analyses were not performed, but we acknowledge that each procedure carries a distinct risk profile. We also recognize that the composite of wound‐related complications pooled mechanistically distinct events including seroma and hematoma, which may obscure true differences in wound‐healing risk.

The cohort was also clinically heterogeneous with respect to cancer type, disease stage, and prior oncologic therapy. These factors may independently influence wound healing and were incompletely captured in this retrospective study. We acknowledge that the two comparison groups were inherently different owing to the differences in underlying malignancy and treatment course. The treatment group had a significantly higher BMI and a higher rate of concurrent steroid use, which may have confounded the observed association. Due to the small number of events, our multivariable model cannot fully separate the independent effect of targeted therapy from that of BMI and steroid exposure. Mean age and the prevalence of prior surgical site infection were also higher in the treatment group; however, these factors did not result in a significant association with wound healing.

Our findings should be regarded as hypothesis‐generating. These limitations overall underscore the need for larger, prospective, procedure‐stratified studies to more precisely characterize wound‐healing risk in cancer pain patients. Importantly, although interventional pain procedures are frequently perceived as less invasive, they nonetheless involve meaningful periprocedural risk, especially in immunocompromised patients. Recognizing these risks is essential to informed patient selection, interdisciplinary perioperative planning, and shared decision‐making.

## 5 Conclusion

Our retrospective cohort study provides hypothesis‐generating findings on wound‐related complications in cancer pain patients receiving targeted cancer therapy. We hope to emphasize the need for more careful periprocedural planning and encourage pain physicians to engage in interdisciplinary discussions with oncologists to optimize patient care. Further investigations in larger, prospective studies are needed to confirm our findings and to better characterize the various factors affecting periprocedural risks in cancer pain patients. While our study focused on wound‐related complications, it is also important to study various hematologic issues, especially for neuraxial procedures. Future prospective studies should also assess the safety of periprocedural cancer treatment cessation and provide recommendations for the periprocedural period. Additionally, future studies should compare these risks between different classes of cancer treatment and between patients with and without cancer.

## Author Contributions

**Soun Sheen:** conceptualization, investigation, writing – original draft. **Saba Javed:** conceptualization, writing – original draft, funding acquisition, validation, methodology. **Vishal Bansal:** conceptualization, investigation, writing – original draft. **Jian Wang:** conceptualization, investigation, writing – original draft. **Xiaowen Sun:** conceptualization, investigation, writing – original draft.

## Funding

The authors have nothing to report.

## Ethics Statement

This study was approved by the MD Anderson Cancer Center institutional review board (#2024‐0319).

## Consent

Written or verbal consents were waived as the study involved no more than minimal risks to the subjects. The data was already available and subjects were not contacted during the study.

## Conflicts of Interest

The authors declare no conflicts of interest.

## Data Availability

The authors confirm that the data supporting the findings of the study are available within the article.
